# The Role of SprIR Quorum Sensing System in the Regulation of *Serratia proteamaculans* 94 Invasion

**DOI:** 10.3390/microorganisms9102082

**Published:** 2021-10-02

**Authors:** Olga Tsaplina, Inessa Khmel, Yulia Zaitseva, Sofia Khaitlina

**Affiliations:** 1 Institute of Cytology, Russian Academy of Sciences, Tikhoretsky av. 4, 194064 St Petersburg, Russia; skhspb@gmail.com; 2 Institute of Molecular Genetics of National Research Center “Kurchatov Institute”, Kurchatov sq. 2, 123182 Moscow, Russia; khmel@img.ras.ru (I.K.); zjv9@mail.ru (Y.Z.); 3 Laboratory of Biotechnology and Applied Bioelementology, Demidov Yaroslavl State University, Sovetskaya Str. 14, 150003 Yaroslavl, Russia

**Keywords:** quorum sensing system, serralysin, outer membrane protein OmpX, protealysin, hemolytic toxin, *Serratia proteamaculans*, bacterial invasion

## Abstract

The bacteria *Serratia proteamaculans* 94 have a LuxI/LuxR type QS system consisting of AHL synthase SprI and the regulatory receptor SprR. We have previously shown that inactivation of the AHL synthase *sprI* gene resulted in an increase in the invasive activity of *S. proteamaculans* correlated with an increased bacterial adhesion. In the present work, the effects of inactivation of the *S. proteamaculans* receptor SprR are studied. Our results show that inactivation of the receptor *sprR* gene leads to an increase in bacterial invasion without any increase in their adhesion. On the other hand, inactivation of the *sprR* gene increases the activity of the extracellular protease serralysin. Inactivation of the QS system does not affect the activity of the pore-forming toxin ShlA and prevents the ShlA activation under conditions of a limited concentration of iron ions typical of the human body. While the wild type strain shows increased invasion in the iron-depleted medium, deletion of its QS system leads to a decrease in host cell invasion, which is nevertheless similar to the level of the wild type *S. proteamaculans* grown in the iron-rich medium. Thus, inactivation of either of the two component of the *S. proteamaculans* LuxI/LuxR-type QS system leads to an increase in the invasive activity of these bacteria through different mechanisms and prevents invasion under the iron-limited conditions.

## 1. Introduction

Invasion of host cells by the facultative pathogen *Serratia proteamaculans* 94 arises at the stationary growth phase when the density of the bacterial population is maximal [1]. In response to changes in the density of the population, the Quorum Sensing System (QS) regulates gene expression involved in controlling the lifestyle and activity of bacteria, including the activity of their virulence factors. *S. proteamaculans* 94 has a LuxI/LuxR type QS system consisting of AHL synthase SprI and regulatory receptor protein SprR. In a classical *Aliivibrio fischeri* [2] LuxI/LuxR QS model, AHLs’ binding stabilize the LuxR-type proteins, allowing them to bind DNA and activate transcription of target genes [3], including the LuxI-type AHL synthase gene. In this QS model, expression of the AHL synthase gene requires AHL activation of the LuxR protein. However, the QS system of *S. proteamaculans* 94 is different from the *A. fischeri* QS system: the AHL synthase *sprI* gene and the *sprR* gene encoding a receptor protein are convergently transcribed and overlapped in their terminal areas [4]. In contrast to the classical QS system of *A. fischeri*, AHL synthesis by the *S. proteamaculans* 94 does not depend on the SprR regulatory receptor protein [5]. Accordingly, only a mutation in the AHL synthase *sprI* gene, but not a mutation in the receptor *sprR* gene, led to a decrease in exoprotease and chitinolytic activities and the ability to form biofilms by *S. proteamaculans* 94 [5].

Previously, we have shown that inactivation of the AHL synthase *sprI* gene resulted in the increase in invasive activity of *S. proteamaculans* preceded by the increased bacterial adhesion to the cell surface [6]. By our data, the increased adhesion of *S. proteamaculans* SprI(-) could be caused by both an increase in the expression of the surface protein OmpX responsible for adhesion [6,7,8] and a decrease in the activity of protease protealysin, whose substrate is OmpX [7,8]. On the other hand, the AHL signaling molecules can directly affect host cells and their multiple signaling pathways by triggering calcium mobilization, activation of Rho GTPases, MAPK, and NFκB that control diverse host cells functions and behaviors, including cytoskeleton remodeling [9]. In epithelial cells, AHL interacts with the GTPase-activating proteins which lead to phosphorylation of Rac1 and Cdc42 [10]. Phosphorylation of these Rho GTPases leads to their activation, and this may be a necessary stage of bacterial invasion [11]. Therefore, bacteria *S. proteamaculans* SprR(-) with an inactivated SprR receptor that does not affect the AHL synthesis have become an excellent model for assessing the direct effect of signaling AHL molecules on the host cell. The aim of the present work is to unravel the effect of inactivation of the regulatory AHL-receptor SprR on the *S. proteamaculans* invasive activity and the activity of known virulence factors.

Our results show that, in contrast to the inactivation of the AHL synthase *sprI* gene [6], inactivation of the regulatory receptor protein *sprR* gene does not lead to an increase in the expression of the surface protein OmpX, which is responsible for adhesion, and does not affect the activity of protease protealysin, the substrate of which is OmpX. Despite the absence of an effect on adhesion, inactivation of the *sprR* gene leads to an increase in the invasive activity of *S. proteamaculans* correlated with the increase in the activity of extracellular metalloprotease serralysin, which is cytotoxic to mammalian cells. In addition, the inactivation of the *sprR* gene as well as inactivation of the *sprI* gene [6] results in a loss of the *S. proteamaculans’* ability to adapt to iron-limiting conditions. This can be a key factor in influencing the spread of infection in the body, because iron content in the body is limited during infections due to sequestration by host proteins such as lactoferrin, transferrin, and haemoglobin. These results indicate that inactivation of both proteins of the LuxI/LuxR-type QS system through independent mechanisms leads to a similar increase in the invasive activity of *S. proteamaculans* and prevents this effect under iron-limited conditions typical of the human body.

## 2. Materials and Method

### 2.1. Cell Cultures, Bacterial Strains, and Growth Conditions

Human cervical carcinoma cells M-HeLa and embryonic mouse fibroblasts transformed with SV40 virus (Balb 3T3-SV40) were obtained from the Vertebrate Cell Culture Collection (Institute of Cytology, St. Petersburg, Russia) supported by the Ministry of Science and Higher Education of the Russian Federation (Agreement №075-15-2021-683). M-HeLa cells were grown in α-MEM (Minimum Essential Medium α) supplemented with 1% NEAA (Non-Essential Amino Acids) medium, and 3T3-SV40 cells were grown in DMEM (Dulbecco’s Modified Eagle Medium). The cells were grown in the antibiotic-free media supplemented with 10% fetal bovine serum (Sigma, Darmstadt, Germany) at 37 °C under 5% CO_2_ atmosphere, for the time required to get 70–90% confluent (usually 18–40 h).

The wild-type *Serratia proteamaculans* strain 94 (*S. proteamaculans* (w.t.)) was isolated as described earlier [12]. *S. proteamaculans* SprR(-) carrying the inactivated receptor gene was obtained by inserting a suicide vector into the *S. proteamaculans* 94 *rif-r* chromosome by homologous recombination using the sacB-based strategy [13], as described earlier [5]. This suicidal vector for the *sprR* gene contained the full-length 750 bp *sprR* gene with the insert 865-bp fragment from the p34S-Gm plasmid bearing the gentamicin resistance [5]. To replace the native sprR gene in *S. proteamaculans* with the defective gene, the vector was transferred to the *S. proteamaculans* strain by conjugation of *S. proteamaculans* and *Escherichia coli* S-17 strains carrying the pEX18Tc plasmid with an inactivated *sprR* gene. Rifamycin resistance was necessary for the subsequent separation of *S. proteamaculans* from *E. coli*. PCR was used to test the insertion of the gentamicin resistance gene into the *sprR* gene. After inactivation of the *sprR* gene, bacteria acquired the gentamicin resistance gene and the size of the *sprR* PCR product increased as a result of the insertion of the gentamicin resistance gene. We have verified that *S. proteamaculans* 94 *rif-r* carrying an additional spontaneous mutation of rifampicin resistance did not have higher invasive activity than the wild-type *S. proteamaculans* 94. Inactivation of the *sprR* gene did not affect the expression of the *sprI* gene (not shown), synthesis of AHL, chitinolytic activity, or swimming mobility [5].

*S. proteamaculans* were grown in Luria broth (LB medium) in the absence or presence of 0.3 mM 2,2′-bipyridyl at 30 °C with agitation speed controlled at 150 rpm for 21 h or 44–48 h until actinase activity on the *S. proteamaculans* extract could be determined [1]. The aliquots of every bacterial culture were diluted with growth medium to the equal optical densities at 600 nm.

### 2.2. Limited Proteolysis Assay

Rabbit skeletal muscle actin used as a substrate for protealysin was isolated by a standard procedure of Spudich and Watt [14]. G-actin in buffer G (0.2 mM ATP, 0.1 mM CaCl_2_, 5 mM Tris-HCl, pH 7.5, 0.02% NaN_3_) was stored as aliquots (1–3 mg/mL) at −20 °C for a single use. To determine the ability of bacterial extracts to cleave actin, bacteria were pelleted by centrifugation at 12,000× *g* for 10 min, the pellets were re-suspended in buffer G, and the bacteria were lysed by seven cycles of freezing and thawing. The bacterial extracts were clarified by centrifugation at 12,000× *g* for 10 min. The clarified bacterial extracts were mixed with an equal volume of actin and incubated for 18 h at 4 °C. The reaction was stopped by the addition of an equal volume of the electrophoresis sample buffer containing 4% SDS, 125 mM Tris-HCl, pH 6.8, followed by a 5 min boiling. The digestion products were analyzed by SDS PAGE [15]. The actinase activity was determined by the appearance of the 36 kDa fragment resulting from the cleavage of actin by protealysin.

### 2.3. Hemolysis Assay

To determine hemolytic activity of *S. proteamaculans*, the standard hemolysis assay with erythrocytes was used [16], with modifications [17]. Horse erythrocytes were a generous gift from Maria Sergeeva (Research Institute of Influenza, Saint Petersburg, Russia). Washed erythrocytes were suspended in PBS to a final concentration of 2%. *S. proteamaculans* were grown for 48 h, and their extracts were tested for the actinase activity [1]. The aliquots of every bacterial culture were diluted with growth medium to the equal optical densities at 600 nm. Bacteria were pelleted by centrifugation at 12,000× *g* for 10 min, resuspended in 100 µL PBS, and the bacteria suspension was added to 1 mL of erythrocyte suspension for 1 h at 37 °C and then centrifuged for 3 min at 2000× *g*. The absorbance of released hemoglobin was measured at 405 nm. Hemolytic activities are presented as the percentage of the total erythrocytes lysed by 4% SDS.

### 2.4. Zymography with Gelatin

The ability of extracellular bacterial protease to cleave gelatin was performed by zymography. Bacteria were grown in the LB medium for 48 h, then the growth medium (conditioned medium) was clarified, incubated with electrophoresis sample solution (62.5 mM Tris–HCl, pH 6.8, 0.1% SDS) for 30 min at the room temperature, and analyzed by SDS-PAGE, which is different from a regular SDS-PAGE due to the presence of 0.3% gelatin in the resolving gel. After electrophoresis, the gel was washed twice with 2.5% Triton X-100 to remove SDS and incubated in 5 mM CaCl_2_, 50 mM Tris–HCl, pH 7.2–7.4, for 18 h at 37 °C to renature the proteases and carry out proteolysis. Finally, the gel was fixed in 25% isopropanol with 10% acetic acid for 30 min and stained with Coomassie brilliant blue G-250.

### 2.5. Proteolysis of Azocasein

The reaction mixture consisted of 100 µL of azocasein (10 mg/mL) in 50 mM Tris–HCl, pH 7.2, and 50 µL of the conditioned media of *S. proteamaculans* (grown for 48 h) was incubated at 37 °C for 60 min. The reaction was quenched by the addition of 200 µL 10% trichloroacetic acid for 10 min at 20 °C. After centrifugation at 12,000× *g* for 5 min, 250 µL of supernatant was mixed with 50 µL 4 M NaOH, and the absorbance was determined at 450 nm.

### 2.6. Fluorescence Microscopy

Bacteria were grown as described above until the actinase activity of *S. proteamaculans* extracts could be detected [1]. Thirty minutes before the experiment, bacteria (1 mL) were pelleted at 9600 g for 10 min; the pellets were resuspended in FITC (1 mg/mL) of the bacterial culture to visualize the bacteria. Bacteria were pelleted at 9600 g for 10 min; the pellets were resuspended in 100 µL DMEM and added to the host cells in a 1 mL fresh portion of DMEM. The host cells and bacteria were co-cultivated at 37 °C in 5% CO_2_ for 3 h. Cells were washed three times with PBS solution at each staining step. The preparations were fixed with 3.7% formaldehyde solution (Sigma, Darmstadt, Germany) for 10 min, incubated for 5 min with 0.1% Triton X100 and stained with rhodamine-phalloidin (Sigma, Darmstadt, Germany) for 15 min to visualize the actin cytoskeleton. The samples were mounted in the mounting medium and analyzed with a confocal fluorescence Leica SP5 TCS microscope using dual 488 nm (green fluorescence) and 543 nm (red fluorescence) laser system to visualize the FITC-stained bacteria and the rhodamine–phalloidin-stained cytoskeleton, respectively.

### 2.7. Quantitative Adhesion and Invasion Assay

Efficiency of invasion and adhesion was evaluated by the quantitative invasion assay [8,18]. Bacteria (1 mL) were pelleted at 9600× *g* for 10 min; the pellets were then resuspended in 100 µL DMEM and added to the host cells in a 1 mL fresh portion of DMEM. After co-cultivating host cells and bacteria at 37 °C in 5% CO_2_ for 1–2.5 h, unattached bacteria were washed out twice with PBS and the infected cells were suspended in 0.25% trypsin–versene solution. To quantify the effectiveness of adhesion, suspension of the infected cells was quickly diluted and plated out on LB agar to determine the number of colony forming units (CFU) of adherent bacteria. To quantify the effectiveness of invasion, suspension of the infected cells was incubated in DMEM containing kanamycin to kill extracellular bacteria and then the cells were lysed with 1.5% sodium deoxycholate, quickly diluted with cold LB medium, and the aliquots of the resulting suspension were plated on LB agar to determine the number of colony forming units (CFU) of intracellular bacteria [8]. The results for each experiment are the average of an assay performed in triplicate and independently repeated three times.

### 2.8. PCR Analysis

Genes’ expression was analyzed by semi-quantitative RT-PCR. Bacteria were grown for 21 h and pelleted by centrifugation at 12,000× *g* for 10 min. Total RNA was extracted from the bacterial pellet using an extraction kit (DiaM, Russia) according to the manufacturer’s instructions. Reverse transcription was performed with the RevertAid First Strand cDNA Synthesis Kit (Thermo Fisher Scientific, Waltham, MA, USA) and the resulting cDNA was diluted in H_2_O to 20 ng/mL. Gene-specific primer pairs designed using BLAST-primer software were as follows: for *ompX* gene, 5′-GCAGTAGCAGCCTGTGTATTA-3′ and 5′- TTGGGCGTTGTCGATGTT-3′; for serralysin gene, 5′-AGGCAACCCAACCTACAA-3′ and 5′-AACCGGAGAAGTCGAAAGT-3′; for protealysin gene, 5′-GGTGAAGTCATCCGCGATATT-3′ and 5′-ATCAGCCAGTCGGCTTTATC-3′; for *ShlA* gene, 5′-GCACAACGGACAGCTACTAT-3′ and 5′-GGATCTCGTCACCCTGAATATC-3′; and for S12 ribosomal protein gene, 5′-CAGAAACGTGGCGTATGTACT-3′ and 5′-CGAGCTTGCTTACGGTCTTTA-3′. The PCRs were optimized independently for each set of primers as follows: primary melting at 94 °C for 3 min, 30 amplification cycles (94 °C for 1 min, annealing temperature of 62 °C for 1 min, 72 °C for 1 min), and a final elongation step of 10 min at 72 °C. Amplified products were resolved on 1% agarose gels and visualized with SYBR Safe (Thermo Fischer Scientific) staining. Gene expression was evaluated relative to the constitutive expression of a S12 ribosomal protein gene. Three biological replicates were analyzed.

### 2.9. Statistical Analysis

Data were analyzed statistically using one-way analysis of variance (ANOVA) with the Excel Data Analysis Pack. A difference was considered significant at the *p* < 0.05 level.

## 3. Results

### 3.1. The Effects of the Receptor sprR Gene Inactivation on the S. proteamaculans Invasive Activity

Previously we have shown that inactivation of the AHL synthase *sprI* gene resulted in a more than fourfold increase in the invasive activity of *S. proteamaculans*, preceded by the increase in bacterial adhesion to the cell surface [6]. To evaluate the contribution of another QS system component, regulatory receptor protein SprR, to the regulation of invasive activity of *S. proteamaculans* 94, we used a *S. proteamaculans* SprR(-) mutant carrying the inactivated receptor *sprR* gene.

**Figure 1:**
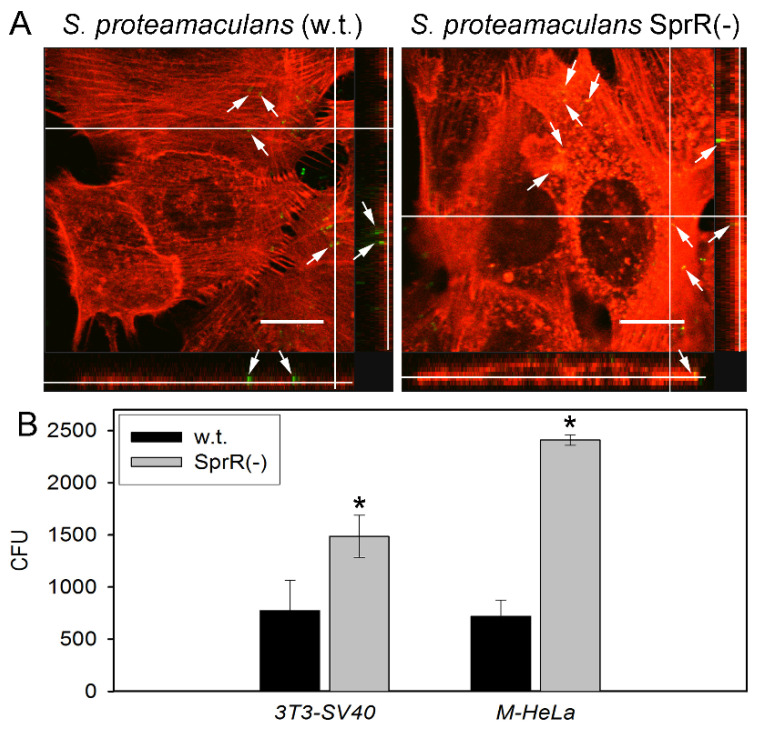
Invasion of cultured eukaryotic cells by the mutant and the wild-type *S. proteamaculans* (w.t.). (**A**) Confocal microscopy images of 3T3-SV40 cells incubated with *S. proteamaculans* (w.t.) and *S. proteamaculans* SprR(-) containing the inactivated receptor *sprR* gene for 3 h. Cytoskeleton was stained with rhodamine phalloidin, bacteria were stained with FITC. Intracellular bacteria are marked with arrows. Bar, 15 µm. (**B**) Quantitative evaluation of the susceptibility of 3T3-SV40 and M-HeLa cells to invasion by *S. proteamaculans* (w.t.) and *S. proteamaculans* SprR(-). The 3T3-SV40 and M-HeLa cells were incubated with bacteria for 2 and 2.5 h, respectively. Values are expressed as mean ± S.D. (error bars). A difference was considered significant at the * *p* < 0.05 level.

By the confocal microscopy of embryonic mouse fibroblasts 3T3 transformed with SV40 virus (3T3-SV40), we showed that inactivation of the receptor *sprR* gene, as well as inactivation of the AHL synthase *sprI* gene [6], does not lead to a decrease in the intensity of invasion (Figure 1A). Using a quantitative invasion assay [18], we compared invasion of human cervical carcinoma cells M-HeLa and 3T3-SV40 cells by the mutant and wild-type *S. proteamaculans* (w.t.). Inactivation of the receptor *sprR* gene led to a two-fold increase in invasion of 3T3-SV40 cells and a three-fold increase in invasion of M-HeLa cells (Figure 1B), which is weaker than the effect of inactivation of the AHL synthase *sprI* gene [6].

### 3.2. The Effects of the Receptor sprR Gene Inactivation on the S. proteamaculans Adhesion to the Eukaryotic Cells

**Figure 2:**
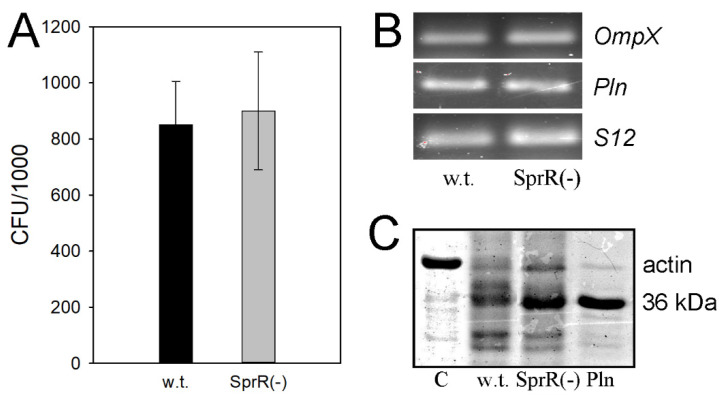
Effects of the receptor *sprR* gene inactivation on the intensity of adhesion of *S. proteamaculans* and the virulence factors regulating the adhesion. (**A**) Quantitative evaluation of the susceptibility of M-HeLa cells to adhesion by *S. proteamaculans* (w.t.) and *S. proteamaculans* SprR(-). The M-HeLa cells incubated with bacteria for 1 h. Values are expressed as mean ± S.D. (error bars). (**B**) Expression of *OmpX* and protealysin (*Pln*) genes in the bacteria grown in LB medium for 21 h, determined by RT-PCR. S12 ribosomal protein served as an internal control. (**C**) The actin-hydrolyzing activity in the extracts of *S. proteamaculans* (w.t.), *S. proteamaculans* SprR(-), and purified protealysin (Pln). C—control, uncleaved actin.

Bacterial invasion consists of two steps: adhesion of the bacteria to the cell surface and penetration of the bacteria into the host cell. Previously, we have shown that the increased invasive activity of *S. proteamaculans* SprI(-) correlates with and could be due to the increased bacterial adhesion to the cell surface [6]. Figure 2A shows that, in contrast, inactivation of the receptor *sprR* gene did not affect the adhesion of the *S. proteamaculans* SprR(-) mutant.

The increase in adhesion of *S. proteamaculans* was previously shown to be associated with an increase in the expression of the bacterial surface protein OmpX [6,7,8] and a decrease in the activity of the bacterial protease protealysin, the substrate of which is OmpX [6,8]. Therefore, we evaluated the effects of the inactivation of the AHL receptor *sprR* gene on the level of the OmpX and protealysin expression. These experiments did not reveal any effects of the SprR(-) mutantion on the OmpX or protealysin expression (Figure 2B). These results were confirmed by a similar actin-hydrolyzing activity in the extracts of *S. proteamaculans* (w.t.) and *S. proteamaculans* SprR(-) (Figure 2C). Taken together, these results suggest that the increase in *S. proteamaculans* invasion upon inactivation of the receptor *sprR* gene occurs during the entry of the mutant bacteria into the host cell. Moreover, other virulence factors can regulate the invasion in *S. proteamaculans* SprI(-) and *S. proteamaculans* SprR(-) mutant strains.

### 3.3. The Effects of the Receptor sprR Gene Inactivation on the Activity of the Toxin ShlA and Serralysin

**Figure 3:**
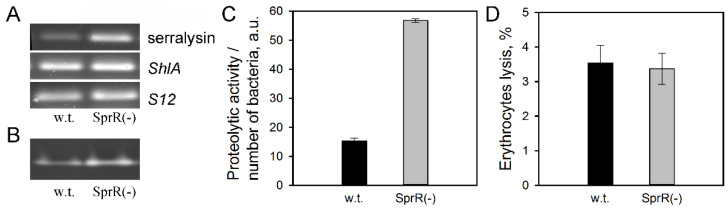
Effects of the receptor *sprR* gene inactivation on the expression and activity of the virulence factors. (**A**) Expression of serralysin and *ShlA* genes in the bacteria grown in LB medium for 21 h, determined by RT-PCR. S12 ribosomal protein served as an internal control. (**B**) The activity of serralysin determined by the gelatin proteolysis in the *S. proteamaculans* (w.t.) and *S. proteamaculans* SprR(-) conditioned growth medium (**C**). The activity of extracellular metalloproteases determined by the azocasein proteolysis in the *S. proteamaculans* (w.t.) and *S. proteamaculans* SprR(-) conditioned growth medium. Values are expressed as mean ± S.D. (error bars). A difference was considered significant at the *p* < 0.05 level. (**D**) The hemolytic activity characterizing ShlA toxin activity in the *S. proteamaculans* (w.t.) and *S. proteamaculans* SprR(-).

Previously, we have shown that the efficiency of the *S. proteamaculans* invasion depends on the activity of the pore-forming toxin ShlA and extracellular protease serralysin [17]. Therefore, we evaluated the effect of the inactivated receptor *sprR* gene on the expression and activity of these virulence factors. Using RT-PCR, it was found that inactivation of the *sprR* gene leads only to an increase in the expression of serralysin (Figure 3A). The serralysin activity was quantified by hydrolysis of azocasein with the total bacterial extracellular metalloproteases and by hydrolysis of gelatin with a 56 kDa metalloproteinase in a bacterial conditioned growth medium. Both methods gave similar results (Figure 3B,C). Inactivation of the receptor *sprR* gene resulted in a more than fourfold increase in proteolytic activity of serralysin (Figure 3C), in contrast to the inactivation of the AHL synthase *sprI* gene, which did not lead to an increase in the activity of serralysin [6]. Thus, inactivation of the AHL synthase *sprI* gene and inactivation of the receptor *sprR* gene leads to an increase in invasive activity through different mechanisms.

**Figure 4:**
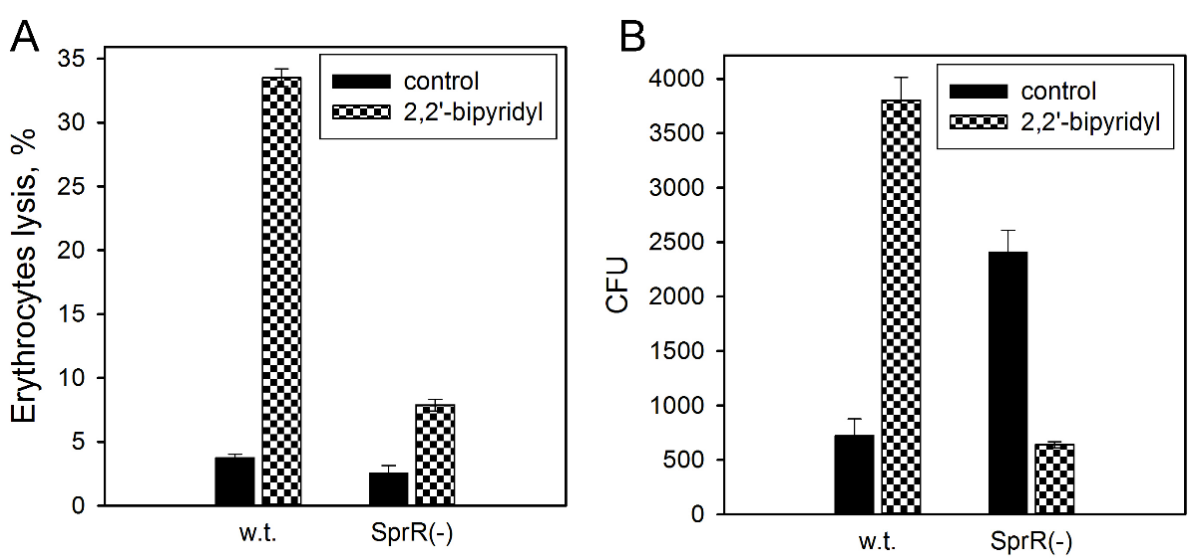
Effects of 2,2′-bipyridyl on the activity of the ShlA toxin and bacterial invasion. (**A**) The effect of 2,2′-bipyridyl in growth medium on the hemolytic activity characterizing ShlA toxin activity in the *S. proteamaculans* (w.t.) and *S. proteamaculans* SprR(-). Control—the ShlA activity of bacteria grown in LB medium without addition of 2,2′-bipyridyl. Values are expressed as mean ± S.D. (error bars). A difference was considered significant at the *p* < 0.05 level. (**B**) Quantitative the effect of bacterial growth in 2,2′-bipyridyl on the susceptibility of M-HeLa cells to invasion by *S. proteamaculans* (w.t.) and *S. proteamaculans* SprR(-). The M-HeLa cells were incubated with bacteria for 3 h. Control—the invasive activity of bacteria grown in LB medium without addition of 2,2′-bipyridyl. Values are expressed as mean ± S.D. (error bars). A difference was considered significant at the *p* < 0.05 level.

The intensity of the bacterial invasion depends also on the activity of the pore-forming toxin ShlA [16,17]. We have shown that inactivation of the receptor *sprR* gene does not affect the activity of the toxin in the LB growth medium (Figure 3D). Previously, we have shown that inactivation of the *sprI* gene resulted in the loss of the ability of *S. proteamaculans* to adapt to iron-limiting conditions [6]. Therefore, the pore-forming toxin ShlA activities in *S. proteamaculans* (w.t.) and *S. proteamaculans* SprR(-) were compared under iron-limiting conditions using an iron chelator 2,2′-bipyridyl. Upon the growth of bacteria in a medium with 2,2′-bipyridyl, the activity of the ShlA toxin in *S. proteamaculans* SprR(-) increases threefold, in contrast to a tenfold increase in the activity of ShlA toxin in *S. proteamaculans* (w.t.) (Figure 4A). Therefore, iron deficiency in the growth medium of bacteria with an inactivated QS system leads to a weaker effect on the activity of the toxin than for bacteria of the wild strain. In the absence of any increase in the toxin activity, the addition of 2,2′-bipyridyl to the *S. proteamaculans* SprR(-) growth medium leads to a 2.5-fold decrease in invasive activity, in contrast to a 5-fold increase in the invasive activity of the wild strain (Figure 4B). These results indicate that inactivation of either of the LuxI/LuxR-type QS system proteins prevents an increase in invasion under iron-limited conditions typical of the human body.

## 4. Discussion

Bacteria coordinate gene expression by quorum sensing (QS) through the production of signal molecules. The N-acyl-L-homoserine lactones (AHL) signal molecules are used by LuxI/LuxR-type QS systems functioning in gram-negative bacteria [19], and the autoinducer-2 (AI-2) signal molecules are used by LuxS-type QS systems in both gram-negative and in gram-positive bacteria [20]. The synthesis of AHL and AI-2 depends on the LuxI and LuxS-type proteins, respectively. QS systems had been demonstrated to play a pivotal role in bacterial pathogenesis by regulating the expression of different virulence factors affecting adhesion, invasion, and survival within a tissue [21]. Strategies aimed at manipulating QS regulation of bacterial virulence are viewed as especially promising. Nevertheless, the effect of the QS system on bacterial invasion, which is a key stage of virulence, has been shown only for LuxS type QS systems and is controversial both for different bacteria and for different host cells. The inactivation of the QS system significantly increased the internalization of *Pseudomonas aeruginosa* [22] and *Streptococcus pyogenes* [23], but reduced the invasiveness of pathogenic *Escherichia coli* (APEC) [24] and *Salmonella* [25]. Inactivation of the QS system of *Campylobacter jejuni* had virtually no effect on invasion in Caco-2 cells [26], but reduced invasion in INT407 cells [27].

*S. proteamaculans* 94 has both the LuxS type QS system and the LuxI/LuxR type QS system consisting of AHL synthase SprI and regulatory protein SprR. Previously published data have indicated that the *luxS* gene inactivation results in a mutant *S. proteamaculans* 94 phenotype are very similar to AHL synthase gene inactivation phenotype [4]. This indicates that both AHLs and AI-2 signal molecules in the *S. proteamaculans* 94 strain could be involved in QS control of the same physiological and metabolic processes. We have shown that inactivation of the AHL synthase *sprI* gene led to a more than fourfold increase in the invasive activity of *S. proteamaculans* as a result of increased adhesion [6]. This increase in the adhesion of *S. proteamaculans* with inactivated AHL synthase gene can be caused by an increase in the amount of the surface protein OmpX and a decrease in the activity of the protease protealysin, the substrate of which is OmpX [6,7,8]. These results allowed us to suggest that synthesis of AHLs controls the invasion of *S. proteamaculans* by accumulation on the bacterial surface of full-length OmpX not cleaved by protealysin [6].

Our present results show that inactivation of the *sprR* gene led to a more than twofold increase in the invasive activity of *S. proteamaculans* SprR(-). Thus, the direct effect of AHL signaling molecules on the host cells is not critical in regulating the intensity of *S. proteamaculans* invasion. Moreover, the results of the present work show that inactivation of the *sprR* receptor gene does not affect the amount of the outer membrane protein OmpX and the activity of protease protealysin cleaving OmpX which, in turn, does not lead to an increase in adhesion. Thereby, inactivation of the *sprR* receptor gene led to an increase in the activity of the extracellular metalloprotease serralysin. Serralysin-like proteases secreted by *S. marcescens* not only suppress cellular immunity by decreasing the adhesive properties of immunosurveillance cells [28], but are also cytotoxic to mammalian cells [29,30,31], thus contributing to bacterial pathogenesis. At the same time, inactivation of the AHL synthase *sprI* gene did not lead to an increase in the activity of serralysin [6]. Thus, inactivation of the *sprI* and *sprR* genes leads to an increase in the intensity of bacterial invasion as a result of the activation of different virulence factors.

The key point for the spread of infection is the regulation of the intensity of the invasion of opportunistic bacteria when they enter the human body. One of the factors signaling the entry of bacteria into the body is a decrease in the concentration of iron in the medium. Because the body’s environmental iron content is limited during infections due to sequestration by host proteins such as lactoferrin, transferrin, and hemoglobin, a decrease in the concentration of iron in the medium leads to the activation of the pore-forming toxin ShlA and an increase in the invasion of *S. proteamaculans* [17]. The pore-forming toxin ShlA not only has a cytotoxic effect on epithelial cells in a culture [32], but is also necessary for invasion by *S. marcescens* [16]. The inactivation of both the *sprI* gene and the *sprR* gene resulted in the loss of the ability of *S. proteamaculans* to adapt to iron-limiting conditions. The iron deficiency in the growth medium of bacteria with an inactivated QS system leads to the decrease in the invasive activity in the absence of an increase in toxin activity. These results indicate that inactivation of either of the LuxI/LuxR-type QS system proteins through different mechanisms leads to the increase in the invasive activity of *S. proteamaculans*, but prevents an increase in invasion under iron-limited conditions typical of the human body.

## References

[1] Tsaplina O.A., Efremova T.N., Kever L.V., Komissarchik Y.Y., Demidyuk I.V., Kostrov S.V., Khaitlina S.Y. (2009). Probing for actinase activity of protealysin. Biochemistry.

[2] Urbanczyk H., Ast J.C., Higgins M.J., Carson J., Dunlap P.V. (2007). Reclassification of Vibrio fischeri, Vibrio logei, Vibrio salmonicida and Vibrio wodanis as Aliivibrio fischeri gen. nov., comb. nov., Aliivibrio logei comb. nov., Aliivibrio salmonicida comb. nov. and Aliivibrio wodanis comb. nov. Int. J. Syst. Evol. Microbiol..

[3] Stevens A.M., Dolan K.M., Greenberg E.P. (1994). Synergistic binding of the Vibrio fischeri LuxR transcriptional activator domain and RNA polymerase to the lux promoter region. Proc. Natl. Acad. Sci. USA.

[4] Zaitseva Y.V., Koksharova O.A., Lipasova V.A., Plyuta V.A., Demidyuk I.V., Chernin L.S., Khmel I.A. (2019). SprI/SprR Quorum Sensing System of Serratia proteamaculans 94. BioMed Res. Int..

[5] Zaitseva Y.V., Lipasova V.A., Koksharova O.A., Plyuta V.A., Demidyuk I.V., Chernin L.S., Khmel I.A. (2021). Peculiarities of the SprIR Quorum Sensing System of Serratia proteamaculans 94 and Its Involvement in Regulation of Cellular Processes. Russ. J. Genet..

[6] Tsaplina O., Khmel I., Zaitseva Y., Khaitlina S. (2021). Invasion of Serratia proteamaculans is regulated by the sprI gene encoding AHL synthase. Microbes Infect..

[7] Tsaplina O.A. (2018). Participation of Serratia proteamaculans outer membrane protein X (ompX) in bacterial adhesion on eukaryotic cells. Tsitologiya.

[8] Tsaplina O., Demidyuk I., Artamonova T., Khodorkovsky M., Khaitlina S. (2020). Cleavage of the outer membrane protein OmpX by protealysin regulates Serratia proteamaculans invasion. FEBS Lett..

[9] Holm A., Vikström E. (2014). Quorum sensing communication between bacteria and human cells: Signals, targets, and functions. Front. Plant Sci..

[10] Karlsson T., Turkina M.V., Yakymenko O., Magnusson K.-E., Vikström E. (2012). The Pseudomonas aeruginosa N-Acylhomoserine Lactone Quorum Sensing Molecules Target IQGAP1 and Modulate Epithelial Cell Migration. PLoS Pathog..

[11] Cossart P., Sansonetti P.J. (2004). Bacterial Invasion: The Paradigms of Enteroinvasive Pathogens. Science.

[12] Demidyuk I.V., Kalashnikov A.E., Gromova T.Y., Gasanov E.V., Safina D.R., Zabolotskaya M.V., Rudenskaya G.N., Kostrov S.V. (2006). Cloning, sequencing, expression, and characterization of protealysin, a novel neutral proteinase from Serratia proteamaculans representing a new group of thermolysin-like proteases with short N-terminal region of precursor. Protein Expr. Purif..

[13] Hoang T.T., Karkhoff-Schweizer R.R., Kutchma A.J., Schweizer H.P. (1998). A broad-host-range Flp-FRT recombination system for site-specific excision of chromosomally-located DNA sequences: Application for isolation of unmarked Pseudomonas aeruginosa mutants. Gene.

[14] Spudich J.A., Watt S. (1971). The regulation of rabbit skeletal muscle contraction. I. Biochemical studies of the interaction of the tropomyosin-troponin complex with actin and the proteolytic fragments of myosin. J. Biol. Chem..

[15] Laemmli U.K. (1970). Cleavage of Structural Proteins during the Assembly of the Head of Bacteriophage T4. Nature.

[16] Hertle R., Schwarz H. (2004). Serratia marcescens internalization and replication in human bladder epithelial cells. BMC Infect. Dis..

[17] Tsaplina O., Bozhokina E., Mardanova A., Khaitlina S. (2015). Virulence factors contributing to invasive activities of Serratia grimesii and Serratia proteamaculans. Arch. Microbiol..

[18] Prouty A.M., Gunn J.S. (2000). Salmonella enterica Serovar Typhimurium Invasion Is Repressed in the Presence of Bile. Infect. Immun..

[19] Bassler B.L. (1999). How bacteria talk to each other: Regulation of gene expression by quorum sensing. Curr. Opin. Microbiol..

[20] Rutherford S.T., Bassler B.L. (2012). Bacterial Quorum Sensing: Its Role in Virulence and Possibilities for Its Control. Cold Spring Harb. Perspect. Med..

[21] Banerji R., Kanojiya P., Saroj S.D. (2020). Role of interspecies bacterial communication in the virulence of pathogenic bacteria. Crit. Rev. Microbiol..

[22] Cao Q., Wang Y., Chen F., Xia Y., Lou J., Zhang X., Yang N., Sun X., Zhang Q., Zhuo C. (2014). A Novel Signal Transduction Pathway that Modulates rhl Quorum Sensing and Bacterial Virulence in Pseudomonas aeruginosa. PLoS Pathog..

[23] Marouni M.J., Sela S. (2003). The luxS Gene of Streptococcus pyogenes Regulates Expression of Genes That Affect Internalization by Epithelial Cells. Infect. Immun..

[24] Palaniyandi S., Mitra A., Herren C.D., Zhu X., Mukhopadhyay S. (2013). LuxS contributes to virulence in avian pathogenic Escherichia coli O78:K80:H9. Vet. Microbiol..

[25] Choi J., Shin D., Ryu S. (2007). Implication of Quorum Sensing in Salmonella enterica Serovar Typhimurium Virulence: The luxS Gene Is Necessary for Expression of Genes in Pathogenicity Island 1. Infect. Immun..

[26] Elvers K.T., Park S.F. (2002). Quorum sensing in Campylobacter jejuni: Detection of a luxS encoded signalling molecule. Microbiology.

[27] Šimunović K., Ramić D., Xu C., Smole Možina S. (2020). Modulation of Campylobacter jejuni Motility, Adhesion to Polystyrene Surfaces, and Invasion of INT407 Cells by Quorum-Sensing Inhibition. Microorganisms.

[28] Ishii K., Adachi T., Hamamoto H., Sekimizu K. (2014). Serratia marcescens Suppresses Host Cellular Immunity via the Production of an Adhesion-inhibitory Factor against Immunosurveillance Cells. J. Biol. Chem..

[29] Marty K.B., Williams C.L., Guynn L.J., Benedik M.J., Blanke S.R. (2002). Characterization of a Cytotoxic Factor in Culture Filtrates of Serratia marcescens. Infect. Immun..

[30] Shanks R.M.Q., Stella N.A., Hunt K.M., Brothers K.M., Zhang L., Thibodeau P.H. (2015). Identification of SlpB, a Cytotoxic Protease from Serratia marcescens. Infect. Immun..

[31] Stella N.A., Callaghan J.D., Zhang L., Brothers K.M., Kowalski R.P., Huang J.J., Thibodeau P.H., Shanks R.M.Q. (2017). SlpE is a calcium-dependent cytotoxic metalloprotease associated with clinical isolates of Serratia marcescens. Res. Microbiol..

[32] Hertle R., Hilger M., Weingardt-Kocher S., Walev I. (1999). Cytotoxic Action of Serratia marcescens Hemolysin on Human Epithelial Cells. Infect. Immun..

